# Greatest Risk Factor for Death from COVID-19: Older Age, Chronic Disease Burden, or Place of Residence? Descriptive Analysis of Population-Level Canadian Data

**DOI:** 10.3390/ijerph20247181

**Published:** 2023-12-15

**Authors:** Susan P. Phillips, Lisa F. Carver

**Affiliations:** 1 Family Medicine and Public Health Sciences, Queen’s University, Kingston, ON K7L 5E9, Canada; 2 Faculty of Health Sciences, Queen’s University, Kingston, ON K7L 5E9, Canada

**Keywords:** COVID-19, aging, geriatrics, chronic disease, multi-morbidity, sex, place of residence, mortality

## Abstract

During the first wave of COVID-19, three-quarters of Canadian deaths were among those age 80 and older. We examined whether age, chronic disease load, sex, or place was the strongest predictor of such deaths. A cross-sectional analysis of administrative data from 1 January 2020 to 30 October 2020 for the population of Ontario *(n* = 15,023,174) was performed. Using logistic regression analysis, we determined whether place of residence (community dwelling, community dwelling with formal home care, or long-term care facility), age group, sex, or chronic disease burden was most strongly associated with the outcome of death within 60 days of a positive SARS-CoV-2 PCR test. Overall, there were 2766 deaths attributed to COVID-19. The age-related odds of dying increased from 6.1 (age 65–74) to 13.4 (age 85 or older) relative to those aged <65 years. This age effect was dwarfed by an odds ratio of 117.1 for those living in long-term care versus independently in the community, adjusted for age, sex, and chronic disease burden. The risk of death from COVID-19 aligned much more with social realities than individual risks. The disproportionate mortality arising specifically from institutional residence demands action to identify sources and ameliorate the harms of living in such facilities.

## 1. Introduction

During the first wave of the pandemic in Canada, approximately three-quarters of deaths occurred among those over age 80, a death rate that was 1016 times greater than that of 20–29-year-old Canadians [1]. This is consistent with data from existing studies that generally identify older age or age combined with multimorbidity as the greatest risk for death from COVID-19 [2,3,4]. Across 42 studies, Dessie et al. found that older age, in particular, as well as being male, a smoker, and having cardiovascular disease, chronic obstructive lung disease, diabetes, hypertension, obesity, or cancer, increased the risk of dying among those with COVID-19 [2]. A second systematic review reported similar findings [3]. Few, however, have examined whether the apparent vulnerability of old age masked an even greater risk associated with the place of residence. There are some observational data suggesting that older adults living in the community, that is, aging in place, even those who needed and were receiving formal home care, did not experience excess mortality [5,6], while those living in long-term care institutions (LTC) were at a disproportionately higher risk of death from COVID-19. For example, there was a 72% increase in deaths in Welsh care homes between March and June of 2020 [7]. This increased mortality was attributed to COVID-19. Similar outcomes were seen in England and Scotland [8,9]. These increases were also presumed to be a result of COVID-19, although a causal relationship could not be proven. Across countries, during the first and second waves of the pandemic, there were press reports suggesting that older adults living in institutional settings (e.g., LTC or nursing homes) were more likely to die after testing positive than were those of similar age who were aging in place. 

LTC, in the Canadian context, generally refers to residential settings providing 24 h support to people with complex health needs, such as assistance with activities of daily living and/or health care [10]. There are over 2000 LTC facilities in Canada, with more than 600 of these located in Ontario, Canada’s province with the largest population. The majority of Ontario LTC facilities (57%) are private for-profit businesses, another 27% are private not-for-profit organizations, with a small proportion (16%) being publicly owned [11]. Despite residents of formal care facilities comprising only 7% of the Canadian population, composite data (as of 30 September 2020) demonstrated that they made up 18% of those infected with and 81% of those dying from COVID-19 [11]. This mortality proportion appears to exceed that of all other Organization for Economic Co-operation and Development (OECD) countries, where the comparable average (for death) was 42% [5,11,12]. Prior to the pandemic, 91% of Canadian LTC residents were over age 65, and 74% were 80 years of age or older [11]. What, then, were the relative contributions of age and place of residence to death from COVID-19 in the Canadian context?

We sought to understand whether age or living place was the key determinant of excess risk. Our aim was, therefore, to identify the separate and relative impacts of age and place of residence (community dwelling or LTC) on the risk of death aligned with a positive SARS-CoV-2 polymerase chain reaction (PCR) test and to do this using administrative data for the entire population of the province of Ontario. Our study also considers the relative contributions of other previously described risks for death from COVID-19, including sex (male/female) and the individual burden of chronic diseases, to the odds of dying from this infection during the first wave of disease in Canada’s largest province.

## 2. Materials and Methods

### 2.1. Setting and Population 

Cross-sectional, population-level, and anonymized cohort data were accessed via the ICES (the Institute for Clinical Evaluative Sciences) Ontario Health Data Platform (OHDP). The OHDP links primary health datasets from a variety of sources including verifiable administrative medical and laboratory data. Information in the OHDP can only be accessed by researchers in a de-identified form (individual Ontarians’ names, addresses, and other identifying information are not released). The ICES is an independent non-profit research institute funded by the Ontario government. As a prescribed entity under Ontario’s privacy legislation, the ICES is authorized to collect and use health care data for the purposes of health system analysis, evaluation, and decision support. Secure access to these data is governed by policies and procedures that are approved by the Information and Privacy Commissioner of Ontario. The use of the data in this project is authorized under Ontario’s Personal Health Information Protection Act (PHIPA) and does not require review by a Research Ethics Board. Datasets used included the Discharge Abstract Dataset (DAD), the National Ambulatory Care Reporting System, the Ontario Health Insurance Plan Claims Database (OHIP), the Registered Persons Database, the Ontario Laboratory Information System (OLIS) COVID-19 Laboratory Data, the Ontario Marginalization Index, the Continuing Care Reporting System, and datasets for specific chronic diseases. Datasets were linked using unique encoded identifiers and analyzed within the ICES/OHDP secure environment. All 15,023,174 Ontarians from newborn to age 85 years and older in the ICES/OHDP database for the study period of 1 January 2020 to 30 October 2020 were included.

### 2.2. Data Availability 

All data used are held securely in coded form at the ICES. While legal data sharing agreements between the ICES and data providers (e.g., healthcare organizations and governments) prohibit the ICES from making the dataset publicly available, access may be granted to those who meet pre-specified criteria for confidential access, available at www.ices.on.ca/DAS. The full dataset creation plan and underlying analytic code can be made available from the authors upon request, with the understanding that computer programs may rely upon coding templates or macros that are unique to the ICES and may therefore be either inaccessible or require modification. Data were accessed repeatedly in July and August 2022 and in January 2023. The authors did not have access at any time to any information that could identify individuals.

### 2.3. Outcome Measure

The outcome measure of interest was the death of anyone in Ontario, Canada, between 1 January 2020 and 30 October 2020 who also had a positive PCR SARS-CoV-2 test prior to 30 September 2020, as indicated in the Ontario Laboratories Information System (OLIS), and within 60 days of dying. Early in the pandemic, OLIS captured 80–85% of those with a SARS Co-V-2 PCR test in Ontario. To account for the known lag time in PCR test recording in OLIS, we included those who died after testing positive between 30 August 2020 and 30 September 2020. This, therefore, included those who tested positive in September 2020 and died between 30 and 60 days after testing. As LTC residents were routinely tested even if asymptomatic, while those living in the community could only access PCR testing if they had clinical signs of COVID-19, that is, those who were already sick, the timing just described might underestimate COVID-19 deaths among LTC residents relative to community dwellers who might have been several weeks further along the trajectory of illness when tested. As just stated, during the first phase of the pandemic, PCR testing was not generally available to those in the community without symptoms but was performed widely on asymptomatic residents of LTC facilities, on all hospitalized patients with possible COVID-19 infection, and those who died in a hospital. It is, therefore, not possible to establish comparable test positivity rates across the three place of residence groups studied. For this reason, in addition to the key outcome of interest being death rather than infection, positive PCR test results are reported but were not chosen as an outcome. 

### 2.4. Independent Variables

The main exposure was the place of residence. A residential setting was categorized as a community dwelling (those living independently in their own residences), a community dwelling with formal home care (living independently but receiving state-funded in-home care for health reasons), or an LTC facility. Other independent variables examined included age group, sex, and chronic disease burden. Age was grouped as under 65, 65–74, 75–84, and 85 years old or older. The chronic disease burden was a tally of the diseases most likely to contribute to death and ranked as 0–4+. These included congestive heart failure, chronic obstructive lung disease, asthma, cancer, and cardiac disorders including angina or ischemia and previous bypass surgery.

### 2.5. Statistical Methods

We first recorded descriptive statistics (frequencies and percentages) for age group, sex, residential setting, and chronic disease load. We then performed Chi-squared analyses to test for the relationships between each independent variable and death from COVID-19. Logistic regression analysis was performed to assess the relative associations between each variable (place of residence, sex, age, and chronic disease burden) and death after a positive SARS-CoV-2 PCR test while controlling for the other independent variables. Logistic regression was chosen since the variable of interest was binary (death from COVID-19) and the observations were independent (there were no repeated measures variables). Individuals who had received multiple PCR tests were only counted once. The large size of the dataset was expected to result in statistically significant results for almost all analyses but also render meaningful odds ratios. We chose to keep outliers in the model. As the proportion of those with 4 or more chronic diseases was small, we combined this category with the preceding one, using the categories of 0, 1, 2, or 3 or more chronic diseases for analyses. As a result, there was no need to suppress any findings, as there were no small cells as defined by the ICES/OHDP.

The Wald test was used to perform a joint test of the null hypotheses. Since *p* values are often significant where the sample size is large, we included the Chi-squared statistic and confidence intervals, where applicable.

All analyses were performed using SAS 9.4 and completed within the secure environment described above. 

## 3. Results

### 3.1. Population Characteristics

The demographic characteristics of Ontario’s population during the study period, loosely referred to as the first wave of COVID-19 in the province, are described in Table 1. From 1 January 2020 to 30 October 2020, 85,076 members of the overall population lived in LTC facilities, 512,093 were receiving formal state-supported care while living at home, and the remaining 14,426,005 were community-dwelling and receiving no state-funded homecare. During the study timeframe, a total of 88,860 members of the whole cohort died from all causes. 

### 3.2. Characteristics of Individuals with Positive PCR Tests

As shown in Table 2, 46,570 individuals had a positive SARS-CoV-2 test between 1 January 2020 and 30 September 2020, 54% of whom were women, and 46% were men.

A comparison of PCR test positivity between residents in LTC and those living in the community showed the former to have a statistically higher test positivity proportion (*p* < 0.0001) (see Table 3). 

### 3.3. Characteristics of Those Dying within 60 Days of Positive PCR Testing

In total, 2766 or 0.02% of all Ontarians died between 1 January 2020 and 30 October 2020 and also within 60 days of testing positive for COVID-19 by 30 September 2020. Table 4 demonstrates that the burden, both in frequency and proportion, of those deaths was disproportionately borne by residents of LTC (*p* < 0.0001). 

Logistic regression was used to examine the independent effects of residence location, sex, age group, and chronic disease burden on the likelihood of death. The results are detailed in Table 5. Given the size of the dataset, all of the independent variables were highly significant; however, the Wald Chi-squared statistics varied considerably for long-term care homes versus other residential settings (*X*^2^ = 6185.5641), being almost 7 times greater than the value for age group (*X*^2^ = 895.8). The regression analysis calculated the odds ratios and measures of association (see Table 5) between the outcome (death from COVID-19 after a positive PCR test) and each independent variable (sex, age group, location, and chronic disease burden). Although older age presented a clear increased risk for death from COVID-19 (OR rising to 13.427 with increasing age), the greatest risk was associated with living in LTC rather than in the community without home care (OR = 117.113). Chronic disease burden (OR = 1.532 for 3+ diseases) and sex (OR = 1.596 for men) were each much weaker predictors of death from COVID-19. Small confidence intervals indicate the high precision of all odds ratios.

## 4. Discussion

Older Ontarians’ risk of dying within 60 days of a diagnosis of COVID-19 was 117 times higher if they lived in an LTC facility rather than independently in the community. In contrast, age alone was a much weaker, although still a statistically significant, predictor of death. Somewhat surprisingly, a greater chronic disease burden was of limited predictive value (OR ranging up to 1.532), as was male sex. The findings in this study are consistent with, although more nuanced than, public health surveillance data for Ontario [13]. Official reports documented that from 15 January 2020 to 2 August 2020, 15.0% of all SARS-CoV-2 positive PCR tests were among LTC residents, and that LTC residents 70 years old and older had higher age-specific case fatality rates but also a lower risk of hospitalization or admission to an ICU, when compared to persons of the same age not residing in LTC [13].

Test positivity rates were also much higher among LTC residents (5.96%) than for those living in the community and receiving homecare (0.065%). Hidden within these statistics, however, is the reality that PCR testing was not widely available within the community during the study timeframe. In contrast, those admitted to a hospital were routinely tested, as were asymptomatic residents of any LTC facility when co-habitants became infected. Because of this, comparing test positivity rates would have no real meaning. We focused, therefore, on the proportion of each population group who were presumed to have died from COVID-19. The vastly greater risk of COVID-19 death associated with living in residential care facilities suggests the paradoxical nature of the term ‘care’ in this context. Our results indicate that the stronger determinant of death from COVID-19 during the first wave was living in a formal residential care facility rather than being over 65 years of age or having multiple chronic diseases. One of the proposed sources of infection in LTC was caregivers entering and exiting these facilities [2,3,4,14]. In Ontario, far more older adults received formal care in the community (i.e., while living at home) than in care facilities. This group was also exposed to caregivers frequently entering their homes. These formal caregivers typically visit numerous patients each day, not unlike their counterparts working within LTC facilities. Controlling for age and chronic disease burden, the community-dwelling care-receiving group was at a higher risk of death from COVID-19 (OR = 9.991) than those living in the community. However, the LTC population’s odds ratio of death compared to that of the community-dwelling non-care-receiving group was 117.113 and was approximately 10-fold greater than the risk for those receiving care in their own homes. It would appear that caregivers as vectors of infection were not the sole, or even the key, explanation for the disproportionate death of those residing in LTC.

In addition to examining residence location, we studied those characteristics most often reported by others as sources of risk for COVID-19 infection and death. Our findings indicate that the greatest risk for death from SARS-CoV-2 in Ontario among the variables included was place of residence. The findings cannot explain why living in an LTC facility was so strongly associated with death from COVID-19 or why age and chronic disease burden were much weaker explanatory variables. Likely there were and are aspects of these communal living facilities and the care, or lack of it, that harmed. Others have suggested that the reasons for the disproportionate risk in congregate living facilities such as LTC may include a failure to attend to patient complexity and to basic infection protocols (e.g., isolation from other residents), understaffing coupled with poor working conditions and pay, poor integration of LTC facilities with the broader medical system, and the nature of ‘for-profit’ care typical of privately owned facilities [14]. All of these should and could be addressed to diminish the unnecessary mortality of those living in residential care.

Our data do show an increasing risk of death with increasing age (although only minimally with number of chronic diseases); however, this is dwarfed by the risk associated with one’s place of residence. To the best of our knowledge, ours is the first study to differentiate between place and age/chronic disease burden in determining the relative impact of each factor on mortality and to demonstrate such an excessive risk arising from residence location.

The picture was similar, although much less pronounced, in European countries. Differences in definitions and methodologies notwithstanding, the profile of mortality for the pandemic parallels our findings that the highest rates of death were among formal care-home residents [5]. Older adults who lived in residential settings (e.g., LTC or nursing homes) were also more likely to die after testing positive than were people of the same age who were aging in place. Nevertheless, earlier reports [8,9] suggested that older adults, and particularly those with co-morbidities, were the group most vulnerable to infection with and death from SARS-CoV-2. 

It is essential for policy makers to consider these results when preparing to address the next pandemic and to improve care for older Canadians. One strategy that could be implemented is to increase support that enables older adults to age in place. Our findings indicate that this would be protective. It would also align with the wishes of the majority of older adults to age in place and remain in their own homes within their communities [15,16,17]. This is a less expensive option for governments and individuals than funding LTC [18]. At the individual level, weathering the pandemic in their own homes provided older adults an increased sense of efficacy and a belief that if they were able to endure COVID-19 isolation they could manage anything—including future homebound scenarios [19].

While this study had the strength of a large, reliable, and population-level set of data, some limitations exist. For example, as stated earlier, with large sample sizes, the *p* values will often be significant. To address this limitation, we included Chi-squared statistics, odds ratios, and confidence intervals, where appropriate. Tracking deaths for less than 60 days after positive PCR testing in September 2020 might have increased the apparent deaths among those in LTC (see methods for more regarding this) relative to those living in the community. Correcting this, however, would have increased the magnitude of the disproportionate mortality from COVID-19 in LTC. The cross-sectional cohort enables an examination of associations between variables but not an inference of causality. Caution is needed in generalizing findings even though the population studied was large and was racially, ethnically, and socioeconomically diverse. While PCR testing has proven to be reliable and accurate, there will likely have been some deaths from COVID-19, where no testing was conducted, and some deaths within 60 days of a positive PCR test that were wrongly attributed to COVID-19. We expect that these shortcomings have had limited impact on the findings but cannot confirm this.

## 5. Conclusions

In summary, living in an LTC facility seemed to present the highest risk for death from COVID-19 during the first wave of the pandemic in Ontario, Canada. Our findings have shown that there was a much smaller, although definitely significant, increase in the odds of dying from COVID-19 with increasing age, among adults living at home and receiving homecare, and, to a minimal extent, among men and anyone with multiple chronic conditions. Given that most older adults express a preference for aging in place and ending their lives in their own homes [19] and given the much higher risk of dying after the diagnosis of COVID-19 if one lived in an LTC facility, the benefit of community-based home care, which enables aging in place, is apparent. As with so many aspects of health and illness, it appeared to be the interplay of social circumstances with individual vulnerabilities, rather than those individual traits alone, that was responsible for older adults’ greatest risk of death following COVID-19.

## Figures and Tables

**Table 1:** Characteristics of the Ontario population *(n* = 15,023,174).

**Age** **(in Years)**	**<55**	**55–64**	**65–74**	**75–84**	**85–94**	**95+**
	10,254,362 (68.26%)	2,108,364 (14.03%)	1,502,030 (10.0%)	798,404 (5.31%)	319,448 (2.13%)	40,566 (0.27%)
**Sex**	**Female**	**Male**				
	7,609,994 (50.66%)	7,413,180 (49.34%)				
**Number of Ontarians by Residence Location +/− Homecare (% of total population)**						
Long-term Care (LTC)		Home + Home Care		Other		Total
85,076 (0.57%)		512,093 (3.41%)		14,426,005 (96.02%)		15,023,174
Female	Male	Female	Male	Female	Male	
56,710 (0.38%)	28,366 (0.19%)	280,483 (1.87%)	231,610 (1.54%)	7,272,801 (48.41%)	7,153,204 (47.61%)	15,023,174
**Number of reported chronic diseases by location (% of all at that location)**						
	LTC		Home w/Home Care		Other	
0	10,665 (12.5%)		256,835 (50.2%)		11,630,000 (80.6%)	
1	41,530 (48.8%)		163,132 (31.9%)		2,511,994 (17.4%)	
2	22,183 (26.1%)		70,331 (13.7%)		266,163 (1.8%)	
3	8807 (10.4%)		19,632 (3.8%)		21,644 (0.1%)	
4+	1891 (2.2%)		2163 (0.4%)		845 (0.0%)	
Column Total	85,076		512,093		14,430,000	

**Table 2:** SARS-CoV-2 infection (positive test) by sex.

Sex	Positive Test Numbers	% of All +Tests
Female	24,953	53.58%
Male	21,617	46.42%
Total	46,570	100%

**Table 3:** SARS-CoV-2 infection (positive PCR test) by location (% of all at location).

LTC Residents		Community Dwelling with Homecare		Community Dwelling without Homecare		Total
No *	Yes	No *	Yes	No *	Yes	
80,003 (94.04%)	5073 (5.96%)	508,783 (99.30%)	3310 (0.07%)	14,384,644 (99.74%)	38,187 (0.26%)	15,020,000
(*X*^2^ (2) = 90,806.4053, *p* < 0.0001)						

* Note—no refers to no positive test and includes all those who were not tested, rather than referring to those who tested negative.

**Table 4:** Frequencies of death after a SARS-CoV-2 positive test by age and location.

Age	Long-Term Care (Total *n* = 5073)	Home Care (Total *n* = 3310)	Other (Total *n* = 38,187)
<65	65	38	141
65–74	165	101	124
75–84	409	168	125
85+	1015	315	100
Total (% dying after positive test within each residence category)	1654 (32.6%)	622 (19.8%)	490 (1.3%)

**Table 5:** Binary logistic regression.

Analysis of Effects			
Effect	DF	Wald Chi-Squared	*p* Value
Age group	3	895.8376	<0.0001
Sex	1	141.0318	<0.0001
Location (LTCH vs. Home with Home Care vs. Other)	2	6185.5641	<0.0001
Chronic disease load	3	46.5759	<0.0001
**Odds Ratio Estimates of Death after a positive PCR Test**			
Effect	Odds Ratio	95% Confidence Intervals *	
65–74 vs. 1_<65	6.118	5.160	7.252
75–84 vs. 1_<65	8.976	7.577	10.633
85+ vs. 1_<65	13.427	11.323	15.923
Sex: M vs. F	1.596	1.478	1.724
Location: LTC vs. Other	117.113	102.919	133.264
Location: Home Care vs. Other	9.991	8.718	11.448
Chronic diseases: 1 vs. 0	1.202	1.098	1.317
Chronic diseases: 2 vs. 0	1.228	1.099	1.374
Chronic diseases: 3+ vs. 0	1.532	1.346	1.744

* All *p* values were significant.

## Data Availability

Data can be requested from ICES.
